# Supplementary material for: Atacama Large Aperture Submillimeter Telescope (AtLAST) science: Gas and dust in nearby galaxies
Source: Open Res Eur. 2025 May 23;4:148. Originally published 2024 Jul 12. [Version 2] doi: 10.12688/openreseurope.17459.2 (PMC12287688; doi:10.12688/openreseurope.17459.2)
Supplement: Peer review history

# Reviewer report: Reviewer response for version 2

**DOI:** 10.21956/openreseurope.21684.r54709

**Published:** 2025-07-23

**Authors:** Yogesh Wadadekar

**Affiliations:** 1 NCRA, Tata Institute of Fundamental Research, Ganeshkhind, Maharashtra, India

This paper presents a compelling and comprehensive science case for

 the Atacama Large Aperture Submillimeter Telescope (AtLAST), focusing

 on its potential to revolutionize our understanding of gas and dust in

 nearby galaxies. The authors have done an excellent job of outlining

 the key scientific questions that AtLAST will be uniquely positioned

 to address and have provided a clear and well-structured overview of

 the proposed surveys and technical requirements. The paper is

 well-written, thoroughly researched, and makes a strong case for the

 transformational capabilities of AtLAST.

 Overall, AtLAST is positioned as something that improves on both ALMA

 and APEX. Good sensitivity (particularly for large angular scale

 features which ALMA resolves out), better resolution than APEX, large

 field of view (much larger than ALMA). This makes it a unique

 instrument for nearby galaxy studies. This aspect has been brought out

 in bits and pieces. Would suggest to consolidate these points in one

 place, perhaps in the introduction or conclusion? Figure 1 is

 important to highlight this, but it is not adequately discussed in the

 text.

 Multiwavelength complementarity with Roman/Euclid/LSST and SKA surveys

 have been mentioned, but that discussion could be expanded to describe

 what new science could be enabled by this.

 Minor comments:

 The terms "diffuse" and "dense" gas are used throughout the paper, but

 their definitions are not explicitly stated. Providing a clear

 definition of these terms, perhaps in terms of gas density, would be

 beneficial for the reader.

 The paper mentions different values for AtLAST's FoV (2 degrees in the

 abstract, 1-2 square degrees in the Introduction, and >=60 arcmin in

 Table 1).  This should be clarified for consistency.

 Caption of Figure 1 should be expanded to highlight the exceptional

 capabilities of AtLAST in this parameter space.

 "The currently most powerful near-infrared space telescope, JWST,

 observed warm, shocked, and/or ionized gas in a tiny part of the Large

 Magellanic Cloud" Provide a citation to support this claim.

 In the introduction or the conclusions, authors may consider

 presenting the present status of the proposal, and if possible, rough

 timelines and cost.

 In section 2.1.2, it might be useful to state explicitly how the 15x

 improvement in resolution wrt Herschel translates to spatial

 resolution at tens of parsec cloud scale, up to what distance.

 It would be good to state how many hours of observing time will be

 available per year. This will place the ~2900 hours required for LMC/SMC survey

 in context.

 It is clear that for the LMC/SMC continuum survey, AtLAST will be in a class of

 its own. Radio/submm surveys with current instruments cannot

 compete. However, complementary Roman surveys will be important. If

 there are any relevant surveys planned with Roman, these can be

 mentioned.

 I am not well qualified to comment on Section 2.2 on magnetic fields.

Is the case presented with sufficient detail to be useful for teaching or other practitioners?

Yes

Is the work clearly and accurately presented and does it cite the current literature?

Yes

If applicable, is the statistical analysis and its interpretation appropriate?

Not applicable

Are all the source data underlying the results available to ensure full reproducibility?

Not applicable

Are the conclusions drawn adequately supported by the results?

Yes

Is the background of the case’s history and progression described in sufficient detail?

Yes

Reviewer Expertise:

Galaxy formation and evolution, Star-formation in and around galaxies, Radio AGN, Astronomical Software development, SKAO Monitoring and Control Software.

I confirm that I have read this submission and believe that I have an appropriate level of expertise to confirm that it is of an acceptable scientific standard.

# Reviewer report: Reviewer response for version 1

**DOI:** 10.21956/openreseurope.18868.r42842

**Published:** 2024-10-04

**Authors:** Pastrav Bogdan Adrian

**Affiliations:** 1 Institute of Space Science, Bucharest-Mǎgurele, Romania

The authors present the science case for a concept - a proposed, largest (to date) aperture, 50-m single dish, multi-beam sub-millimeter telescope. The uniqueness of AtLAST comes from its high-resolution, high sensitivity, mapping speeds, very large field of view and large frequency coverage. The need for such an instrument and for an extra-large sub-millimeter survey in the local Universe is clearly emphasized multiple times throughout the paper. The authors focus on four main science areas where AtLAST will be a game-changer for studies of nearby galaxies and not only. All these main science areas are well put into context, with the main questions that exist at present and the technical limitations of current similar surveys (e.g. ALMA, APEX or others) clearly underlined and well documented. The proposed role of AtLAST in shading light in each of these science areas and on the main scientific questions is afterwards described at length.

 The paper is surely worth indexing and will be of interest for the relevant scientific community.

 My comments below are rather minor, with a few points that I would recommend the authors to address (marked with an "*" below).

 Section 1

 1st paragraph: "...applicable in all environments and cosmic times, would arguably have..." -> "...applicable in all environments and at all cosmic times, would arguably have..."

 3rd paragraph - comma after "NOEMA" and "is emerging"

 4th paragraph - "...through both its large aperture, extended 2 degree field of view,..." Here and in the abstract the expected FOV of AtLAST is mentioned to be 2 degrees, in the notes of Table 3 it appears as "1-2 square degrees", while in Section 2 an expected 1 deg FOV is mentioned throughout the various subsections. Could you please clarify

 this for consistency throughout the paper?

 Section 2

 2.1

 2.1.1  Caption of Fig. 1: Instead of left/right -> up /down

    (*)   3rd paragraph, first two sentences - The full range of environments within LMC may very well not span the full range of environments encountered in distant galaxies, for which observations are currently available. Therefore, a quantification of the relative contribution of the various ISM components/tracers to the integrated, unresolved ISM observations, based solely on LMC observations (or even including the SMC and Milky Way) may not offer an accurate enough quantification to be further extrapolated and used uniformly on large scale studies of more distant galaxies.

 I would rephrase a bit these sentences or at least comment upon this issue (or give references if indeed the LMC, SMC & MW present the full range of ISM environments).

 Figure 2 - the last two bottom plots have very blurry axis labels and info, that cannot be distinguished clearly. Maybe you could please fix this minor issue.

 2.1.2 Page 9, last paragraph -> how will the shape of the dust spectral energy distribution from FIR to sub-mm be characterized (e.g. SED model/fitting routine, algorithm or such) to extract the desired parameters? Maybe you can mention this here or in another relevant place;

 (*) Same paragraph: my concern is that studying thoroughly the dust emission only in the LMC and SMC, even though the metallicity regimes are quite different in these two galaxies, will not be enough/sufficient for an accurate understanding of the variation of dust properties with environment and for obtaining better dust properties. Thus, I seriously doubt that "the most precise dust temperature, emissivity index β, and dust masses" will be derived as the authors affirm in this paragraph, based solely on the LMC and SMC study. As the degeneracy between dust temperature and dust emissivity index β is well known, how will this be overcome?

 Shouldn't more nearby galaxies also be included for this purpose (from the Local Group or other nearby ones), that will span a larger range of environments and properties? A few phrases could be added here to strengthen this affirmation and motivate this choice.

 2.2

 2.2.1 3rd paragraph (page 14), "...to accurately estimate the magnetic field direction along the LOS." -> please expand LOS (line-of-sight) as here it is the first time that it is used in the paper

       page 15, end of 1st paragraph: "Multi-wavelength polarimetric observations is required..." -> "Multi-wavelength polarimetric observations are required..."

 2.2.3 1st paragraph , "polarization studies simultaneously will provide very..."-> "polarization studies will simultaneously provide very..."

       2nd paragraph, "each galaxy will be resolved at scales 20 − 300 pc..."-> "each galaxy will be resolved at scales of 20 − 300 pc..."

       3rd paragraph, "Starforming"-> "Star-forming"; last sentence - The information about the required time is missing here "Thus, we estimate the required observing time to map...". Please add this information here. If the estimated observing time(s) is(are) the one(s) from paragraph 5 (the last of this subsection), then this sentence should be removed from this paragraph, as it is not really connected with the previous one(s).

      4th paragraph: "...then examine the emission at the outer disk..." -> "...then examine the emission in the outer disk..."

 2.3

 2.3.2 page 16, 3rd paragraph. The sentence "The Herschel space observatory opened ... should end after the enumeration of references. Then the next one can follow: "However, with 30-40" resolution..."

      last paragraph, page 17 (before section 2.3.3) "Simulations and theory...can vary significant with galactic environments..." ->"...can vary significantly with galactic environments..."

 2.3.3 2nd paragraph, the sentence "In the case of an IFU... " Please delete "thus" from the middle of the sentence and rephrase a bit, e.g. "In the case of an IFU the field of view is much larger, allowing for these nearby galaxies to be covered in one pointing ...."

      Same paragraph, last sentence, in brackets "...(although a multi-beam receiver will enable kinematic studies thus can add additional value)" - > "thus" is also inappropriately used here, as in a few other places in the paper.  -> maybe rephrase as "...(although a multi-beam receiver would enable kinematic studies that would add additional value)".

 2.4

 2.4.1 1st paragraph: "...and how the gas and dust is influenced by..." -> "...and how the gas and dust are influenced by..." ; other references could also be added, from other groups of research (not mainly the ones of some of the authors of the present paper)

 End of 2nd paragraph, page 23: "...of current scaling relations and so provide more reliable constraints..." -> "...of current scaling relations and to provide more reliable constraints..."

 End of 3rd paragraph (page 24), last sentence: "...and mainly located within giant molecular clouds thus much less sensitive to external..." -> "...and mainly located within giant molecular clouds, being much less sensitive to external..."

 2.4.2 3rd paragraph (page 25), first two phrases -> some references seem to be missing here for "large systematic molecular gas surveys of the past 15 years" and for the studies giving an "overview of the basic scaling relations" between the mass of H2 and other galaxy properties. Please add these references.

 2.4.3 1st paragraph - "This consists of two types of targets: a mass-complete sample of field galaxies..." What would be the desired / expected stellar mass range for the sample of field galaxies? It would be worth mentioning this detail here.

 2nd paragraph The phrase "Assuming that the IFU..." is full of information but a bit unclear in its abrupt ending and could also be connected with the following one (but that would create an extremely large sentence). Therefore, please rephrase it so that its ending to have meaning.

 (*) Same paragraph. From previous sections it follows that AtLAST will surely have an 1deg^2 FOV. Here it looks like this an assumption rather than just something very probable. Is the ~1deg^2 field of view achievable and therefore a certainty? Maybe you could clarify this.

 "Observing...thus is better than observing"-> "Observing...therefore being better than observing"

 Section 3

 3.1 - Maybe comment upon why a multi-chroic camera would increase the efficiency of observations; couldn't such a camera be incorporated from the very beginning if this would ensure an increase in sample sizes?

 "larger bandwidths will likely be technically possible across..." -  up to which values (taking into account the limitations due to atmospheric transmission)?

 Section 4, 2nd paragraph.

 "...in our understanding of nearby galaxies are its the sensitivity to extended, low surface brightness emission..." -> "...in our understanding of nearby galaxies are its sensitivity to extended, low surface brightness emission..."

 "...and in each case discuss possible surveys..." -> ...and in each case discussed possible surveys...

Is the case presented with sufficient detail to be useful for teaching or other practitioners?

Yes

Is the work clearly and accurately presented and does it cite the current literature?

Yes

If applicable, is the statistical analysis and its interpretation appropriate?

Not applicable

Are all the source data underlying the results available to ensure full reproducibility?

Not applicable

Are the conclusions drawn adequately supported by the results?

Yes

Is the background of the case’s history and progression described in sufficient detail?

Yes

Reviewer Expertise:

Galaxy structure and morphology, Dust and ISM Physics, Galaxy scaling relations, Galaxy Evolution

I confirm that I have read this submission and believe that I have an appropriate level of expertise to confirm that it is of an acceptable scientific standard.

# Reviewer report: Reviewer response for version 1

**DOI:** 10.21956/openreseurope.18868.r42490

**Published:** 2024-09-23

**Authors:** Matilde Mingozzi

**Affiliations:** 1 Space Telescope Science Institute, Baltimore,, MD, USA

I reviewed the manuscript “Atacama Large Aperture Submillimeter Telescope (AtLAST) science: Gas and dust in nearby galaxies'' by D. Liu and collaborators. This work presents a very detailed review of gas and dust in nearby galaxies and discusses multiple interesting science cases for the new facility AtLAST, a concept for the next generation of 50-m class single-dish observatories. The science background and science cases are clearly presented and it is clearly explained how sub-mm and mm facilitates are crucial to understanding star formation and ISM physics. The paper reads well and is very informative, providing a great overview of how AtLAST could impact galaxy evolution and star formation studies. However, the uniqueness of AtLAST could be better highlighted, as well as its synergy with state-of-the-art and future facilities. Overall, the paper surely deserves to be accepted in Open Research Europe 2024, but I would have some comments, listed below, that I would advise to (partly) incorporate in the final version of the paper. Comments refer to the section numbers and pages of the submitted version of the paper. 

 MAIN GENERAL COMMENTS

1. In many parts of the paper, some gas/galaxy properties are described qualitatively with “generic” adjectives. In this kind of paper, that aims at proving that current facilities are not sensitive enough/do not have enough spatial/spectral resolution and sky coverage, it is particularly important to clarify quantitively properties and instrument characteristics. I have highlighted examples of where the information given could be more quantitative in the minor comments section below. The text should be checked carefully to see where it is more appropriate to implement these comments. An alternative solution would be to move Section 3 (technical requirements) before Section 2 and explain there in detail AtLAST modes (i.e., IFU, multi-beam receiver, etc) and properties (i.e., sensitivity, FOV, spectral resolution, spatial resolution, ..), showing also how they compare with similar current facilities. Then, in the following sections, all the technical details would not need to be repeated because citing this technical section would be enough.
2. Following up on point 1, in order to maximize the importance of the role of AtLAST, a summary table collecting AtLAST characteristics and how they compare to current facilities such as ALMA (possibly also to past facilities) related to the proposed science cases could be added at the beginning/end of a proposed science case section. For instance, for the SMC/LMC science case, this could be done by adding information in Tables 1, 2 and 4, highlighting what is still missing and the level of sensitivity/resolution etc that AtLAST could provide. In this case, the improvement of AtLAST with respect to previous facilities becomes more clear only at the beginning of Section 2.1.3, where for the first time AtLAST capabilities for this science case are specified quantitatively, but this should be better highlighted, given all the already available data discussed in 2.1.1 and 2.1.2.
3. A thing that is currently missing from the paper is a discussion about synergy with state-of-the-art and future facilities. For instance, JWST and Roman are mentioned in figure captions, but never in the text, while SKA and Euclid are mentioned few times. JWST/MIRI (despite the small FOV) can be used to study CO-dark gas using H2 rotation lines in the MIR (e.g., Hernandez+23), but also to study dust with MIR features in the nearby universe. Roman will also be an IR mission, similar to Euclid. Moreover, the paper focuses on science cases mostly related to the local universe, but studying metal-poor environments and low-mass galaxies can be useful to understand properties of high-z systems, mainly studied with HST, ALMA and currently with JWST. Overall, discussing (e.g., in a dedicated section before the conclusions) the synergy between AtLAST and these important current and future missions, and discussing how the presented science cases can be linked to the high-z Universe would help to link even better AtLAST to the big picture.

MINOR COMMENTS

 Introduction:

1. Paragraph 2, page 5 (see also main point n.1): the meaning of “highest physical resolution possible” referred to the Milky Way could be clarified.
2. Paragraph 2, page 5: It is written that “… JCMT and SOFIA are proving …”. However, SOFIA was dismissed so this should be clarified. This is mentioned only later in the paper, so perhaps here “are proving” could just be changed to “proved”. Similar changes could be applied in other parts of the text where SOFIA is mentioned.
3. Paragraph 4, page 5 (see also main point n.1): “unique resolution” is mentioned among the characteristics of AtLAST, but it could be clarified if the term resolution refers to spatial, spectral or both.

Section 2:

1. Some concepts are either briefly mentioned and then explained only much later in the paper or not fully explained, but should be clarified. For instance, the concept of CO-dark gas is first mentioned when describing LMC and SMC in Section 2.1.1, page 7, but it is explained only at the beginning of page 10, in Section 2.1.2. Giant molecular clouds are also mentioned on page 7 for the first time, but it is not mentioned which spatial scales they have, and thus which spatial resolution is needed to study them. Gas and dust tracers are presented in Tables 1 and 2, but it is explained only later in Sec. 2.1.2 why these are good tracers and in which conditions. A possible solution could be to revise the structure of Sections 2.1.1 and 2.1.2, starting by presenting the general background first (integrating parts of Section 2.1.2 in Section 2.1.1) and moving all the details about SMC and LMC after what is written in the current Section 2.1.3, that starts highlighting why AtLAST is really important to study them.
2. Other things mentioned but not well defined are “diffuse” and “dense” gas (e.g., which density and spatial extension?).  

Section 2.1.1:

1. Paragraph 1, page 5: a reference could be added where Figure 1 is mentioned.
2. Paragraph 2, page 5: a reference for 20-50% Zsun for SMC and LMC could be added.
3. Figure 1 caption: “top” and “bottom” panels instead of left and right. Also, the VISTA survey is shown in the bottom panel, as mentioned in the caption, but never in the text. Thus, it is not clear the purpose of the bottom panel of Figure 1.
4. Figure 2 is a bit small and difficult to read.
5. Tables 1 and 2 present an overview of observations of LCM and SMC “hot” and “cold” ISM. However, this is just a sub-selection of information, since LCM and SMC have also been observed with other facilities (e.g., HST, JWST, ESO facilities like MUSE). Here, the reasons for this selection could be clarified (e.g., are these observations mapping entirely LMC and SMC, while others mapped only part of them?). Also, the spatial resolution as well as sensitivity information could be added to these tables for all the listed observations (at the moment spatial resolution in arcsec and pc is specified only for a few of them), to better highlight what is still missing that AtLAST could do. Finally, it could be specified which tracers were used to observe dust in the works listed (e.g., ~70-500 micron with Herschel; dust emissivity index; etc).
6. Paragraph 5, page 7 (see also main point n.1): the meaning of “too large” dust mass and “excessively large dust masses are also observed in high redshift” could be clarified, also specifying which facilities were used at high-z. Also, Rowlands+14 study galaxies at z~1-5, while here z~>6 galaxies are mentioned.
7. Paragraph 6, page 8 (see also main point n.1): the meaning of “sub-mm and mm emission excesses” could be clarified as well as in which nearby galaxies these excesses have been found, apart from SMC and LMC.
8. Paragraph 6, page 9 (see also main point n.1): the resolution needed to “resolve individual environments and account for diffuse dust emission” could be specified, as well as how it is defined the “diffuse gas emission”.

Section 2.1.2:

1. Paragraph 1, page 9 (see also main point n.1): ALMA Largest Angular Structure (LAS), ALMA and APEX, sensitivity, spatial resolution and mapping efficiency could be clarified and compared to the values expected from AtLAST, to let the reader understand the meaning of “poor” and the problem of filtering. In this paragraph, the spatial resolution needed to best study GMCs in SMC and LMC could be specified.
2. Paragraph 2, page 9 (see also main point n.1): the required “higher spatial resolution measurements” could be specified.
3. Paragraph 4, page 10 (see also main point n.1): when discussing CO-dark gas, it is written that CO clumps are “very difficult to detect without sensitive, high resolution and large field-of-view observations”, but this could be explained quantitatively. Also, there have been works studying metal-poor local dwarfs with ALMA, IRAM and NOEMA (e.g., Hunt+14, Hunt+15, Amorin+16, Shi+16, Hunt+17, Cormier+17, Zhou+21) – these works definitely have limitations due to the sensitivity, but it would be interesting to see how AtLAST would improve this.
4. Paragraph 4, page 10 (see also main point n.1): instead of “ALMA’s field of view is too small” and “APEX does not offer enough sensitivity and resolution”, it could be specified what are ALMA FOV and APEX sensitivity and resolution at [CI] frequencies.  
5. Paragraph 5, page 10: “While we could hope that such processes scale linearly with the amount of metals available, studies of nearby galaxies have shown that reality is more complex.” This could be rephrased in “Studies of nearby galaxies have shown that such processes do not scale linearly with the amount of metals available.”
6. Paragraph 5, page 10: it is mentioned a critical metallicity threshold of 0.2 Zsun and then it is suggested that studying LMC and SMC could help to better understand the relation between physical properties and metals. However, LMC and SMC have 0.2-0.5 Zsun, so this point is not very clear.

Section 2.1.3:

1. Paragraph 2, page 10: CMB acronym is defined after mentioning CMB.
2. Paragraph 4, page 12: it is mentioned that ~2900 hours of AtLAST would be needed to map SMC and LMC. Would it be realistic to have ~121 days dedicated to this science case? It would also be interesting to understand how long would it take for current facilities to complete one AtLAST pointing. Also, it is written that “… H2O percentiles of 70, 40 and 10 for the aforementioned bands, we obtain an on-source integration time of 27.0 h, 5.2 h, 28.5 h, respectively” – is there a typo or at 400 GHz only 1/5th of the time is needed?

Section 2.2.1:

1. Last paragraph, page 15  (see also main point n.1): “ALMA can help to some extent in those efforts” could be presented more quantitatively. ALMA is mentioned only once in the previous paragraphs.

Section 2.2.2:

1. Paragraph 1, page 16  (see also main point n.1): Table 3 could be cited when mentioning that the ALMA field of view is not sufficient. Also, AtLAST “super sensitivity” could be expressed quantitatively. Which value is needed for this kind of observations?
2. Paragraph 5, page 16: as commented for section 2.1.3, it would be interesting to read how much would it take for current facilities to do a similar study.

Section 2.3.1:

1. Paragraph 2, page 17: Concerning the KS relation, it has been shown that the law works also for atomic gas, but taking into account the volume density instead of the surface density, suggesting that the atomic gas can be a good tracer of the cold star-forming gas, especially in low-density HI-dominated environments (see e.g., Bacchini+2019).

Section 2.3.2:

1. Paragraph 2, page 18  (see also main point n.1): It is written that “high spatial resolution” means hundreds of pc, while in the previous sections (but also on page 20 when referring to AtLAST’s spatial resolution) the term “high spatial resolution” refers to GMC-scale resolution, so tens of pc or lower. Also, it is written that the resolution of a sub-mm 15-m single dish is insufficient, but this could be defined quantitively (e.g., give an example of resolution in arcsec and in pc for a nearby object, e.g., NGC253 shown in Fig.6, of AtLAST versus current facilities). Also, there are larger single-dishes than 15-m apart from LMT (e.g., IRAM), as written in other parts of the paper.
2. Table 5  (see also main point n.1): There is a “resolution” column, but it is not specified which instrument. The caption could be more specific. Also, it could be useful to specify the typical luminosity of the listed denser gas tracers with respect to CO. Definitely a higher sensitivity is needed to detect them, which could be another aspect in favour of AtLAST.
3. Paragraph 3, page 18  (see also main point n.1): the sentences “extended emission is filtered out” and “ALMA has a very small field of view” could be more specific, for instance giving a range of ALMA LAS and FOV.
4. Paragraph 3, page 18  (see also main point n.1): in the sentence “TP antennas are far from being sensitive enough” the sensitivity of the TP could be specified.
5. Paragraph 5, page 20: on page 13 it is written that AtLAST mapping speed is “comparable with contemporary facilities”, while from here it seems that it is better, so this should be clarified.
6. Paragraph 7, page 20: mapping higher J CO transitions with ALMA is “very difficult” also due to the reduced transmissivity of the atmosphere at higher frequencies, which is true also for future sub-mm facilities, so a further sentence to clarify why things for AtLAST will be easier could be added here.

Section 2.3.2:

1. Paragraph 2, page 21: here it is mentioned for the first time a multi-beam receiver or integral field unit (IFU). It could be clarified why these types of instruments were not needed for the two previous science cases. Also, there are currently no sub-mm IFU, so it would be interesting to know more about this mode (e.g., feasibility, spatial resolution – the spectral resolution and FOV are mentioned/shown). If there are technical AtLAST papers about this topic, they should be cited here. Finally, it is not explained why the spectral resolution of the IFU would be much lower than the one of the multi-beam receiver (for which the spectral resolution is not specified). The IFU is mentioned again in section 2.4.3 on page 24, where it is assumed that the spectral resolution can be higher than what is stated here, so this should be clarified.
2. Last paragraph, page 22: here it is explained how long it would take to observe 100 galaxies with AtLAST for all the transitions, taking as a reference a galaxy like NGC3627. Is the so large difference between this case (~3.5 hr for all the transitions) and the LMC/SMC (>20 hrs) due uniquely to the difference in gas-phase metallicity?

Section 2.4.1:

1. Paragraph 3, page 23: here the topic discussed is the few molecular gas studies of low-mass galaxies (<10^9 Msun), where CO-dark gas can be dominant. It is not clear the link between the first two paragraphs and the third one, which instead discusses cluster environment (and cluster galaxies generally have larger masses and metallicity with respect to less dense environments). Concerning the study of molecular gas in cluster galaxies affected by ram-pressure stripping there are some recent APEX and ALMA studies from the GASP survey (e.g., Moretti+2020, Bacchini+2023).

Section 2.4.2

1. The paragraphs “Molecular gas scaling relations in the dwarf galaxy regime” and “Why do galaxies stop forming stars?” repeat concepts already discussed in 2.4.1 and could be removed/integrated with the text in the previous section.

Section 2.4.3

1. Also in this section, there are repetitions of concepts discussed in 2.4.1 (e.g., 950 hrs of IRAM).
2. Paragraph 2, page 24: Concerning the use of AtLAST for blind surveys including low-metallicity galaxies, it is not clear if it would be feasible to easily detect molecular gas in metal-poor environments. It is not clear if the dependence of CO flux as a function of metallicity (as well as SFR; see e.g., Hunt+2020, MAGMA survey) is taken into account in the calculations presented here, where only 30 mins per source are considered, given the large amount of time needed for one pointing on the SMC/LMC (>20 hrs). Also, here is mentioned a range in redshift of z~0-0.16, while in the previous paragraph, there is written z~0.01-0.38.

Is the case presented with sufficient detail to be useful for teaching or other practitioners?

Partly

Is the work clearly and accurately presented and does it cite the current literature?

Yes

If applicable, is the statistical analysis and its interpretation appropriate?

Not applicable

Are all the source data underlying the results available to ensure full reproducibility?

Yes

Are the conclusions drawn adequately supported by the results?

Partly

Is the background of the case’s history and progression described in sufficient detail?

Yes

Reviewer Expertise:

My area of research is focused on galaxy evolution and interstellar medium studies in the nearby universe. In particular, I am an expert in spectroscopy studies of the ionized gas in different types of galaxies, from massive galaxies with/without AGN activity to metal-poor nearby dwarfs, considered high-z analogs. I have been also involved in studies on the cold molecular reservoirs in nearby luminous IR galaxies and objects affected by ram-pressure stripping, and I am currently carrying out IR and subm-mm studies on metal-poor nearby dwarfs, using JWST/MIRI, IRAM and ALMA observatories.

I confirm that I have read this submission and believe that I have an appropriate level of expertise to confirm that it is of an acceptable scientific standard, however I have significant reservations, as outlined above.
